# Supplementary material for: Common Cations Are Not Polarizable: Effects of Dispersion Correction on Hydration Structures from Ab Initio Molecular Dynamics
Source: J Phys Chem Lett. 2023 May 4;14(19):4403–8. doi: 10.1021/acs.jpclett.3c00856 (PMC10201575; doi:10.1021/acs.jpclett.3c00856)
Supplement: Supplementary file 1 — jz3c00856_si_001.pdf [file jz3c00856_si_001.pdf]

Supporting Information for: Common Cations are not Polarizable: Effects of Dispersion Correction on Hydration Structures from Ab Initio Molecular Dynamics

Vojtech Kostal, Philip E. Mason, Hector Martinez-Seara,* and Pavel Jungwirth*

Institute of Organic Chemistry and Biochemistry of the Czech Academy of Sciences, Flemingovo nám. 2, 166 10 Prague 6, Czech Republic

E-mail: hseara@gmail.com; pavel.jungwirth@uochb.cas.cz

Contributions of the D3 and D4

In Figure S1, we show gas-phase interaction energies of a cation with water (left panel) and the same cation (right) obtained according to the Equation 2 in the main text for Li^+, Na^+, K^+, and Ca^{2+}. E_{int} calculated by the revPBE density functional and the CCSD(T) (in the case of calcium) method are compared on the left-hand side y-axis, while the isolated D3 and D4 dispersion correction to the revPBE energies on the right-hand side y-axis. It is worth a note that we subtracted Coulomb potential from the cation–cation curves on the right-hand side panels of Figure S1 in order to facilitate the readout of features that would be otherwise covered by the charge–charge repulsion.

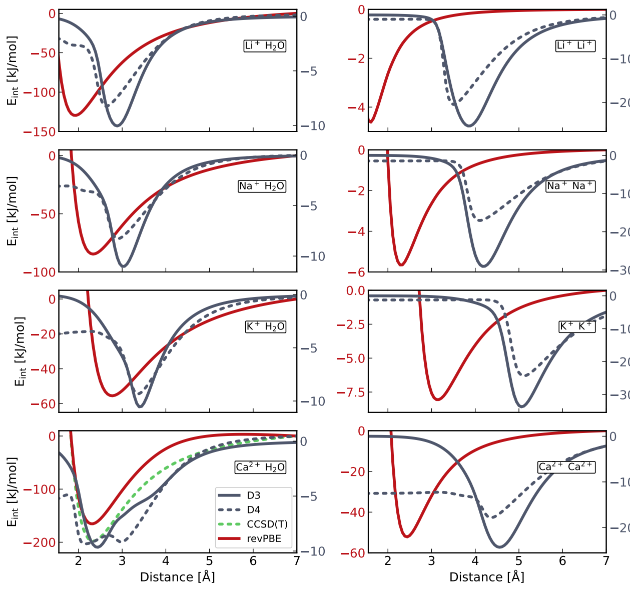

Figure S1: E_{int} of cation–water (left) and cation–cation (right) as a function of the distance for lithium, sodium, potassium, and calcium cations from top to bottom. E_{int} obtained at revPBE level is plotted on the left y-axis in red and the D3 (solid) and D4 (dashed) dispersion correction on the right y-axis in grey. Additional CCSD(T) (dashed green line) result is displayed for calcium cation. Note that Coulomb interaction potential was subtracted from the cation–cation curves.

The D3 contributes to the E_{int} at distances larger than where the minimum is located, which agrees with the results presented in Figure 1 of the main text. This contribution amounts roughly to 10 kJ/mol for cation–water curves. For the cation–cation cases, it increases to 20–30 kJ/mol. The magnitude of the D4 dispersion correction is generally smaller than that of D3. However, the problematic position of the peak is practically the same for both corrections in spite of D4 reflecting better varying electronic structure of the involved species with respect to D3. In the case of calcium, used here as an example of a divalent cation, we see a significant discrepancy between E_{int} calculated either with revPBE and the CCSD(T). The difference is a consequence of the spurious overdelocalization of electrons in the gas phase, which is not surprising for the GGA DFT. This artifact does not occur at the CCSD(T) level.

Additional RDFs of water and chloride anion are shown in this section for the infinitely diluted and concentrated systems in Figure S3. It reveals that neither structure of water nor the hydration of chloride anion is perturbed by the exclusion of metal cation from the D3 dispersion calculation.

Calcium cation at infinite dilution

We simulated a single calcium cation at infinite dilution according to the protocol in the Computational Details of the main text. We extracted the Ca–O RDF and RCN with and without the D3 correction applied to the calcium cation. Both setups yielded similar results, with the first peak at 2.43 Å. This agrees well with the value of 2.38 Å obtained in the neutron scattering experiment.[S1] The main difference between the two simulations is the average CN. In the case where the D3 correction was ap-

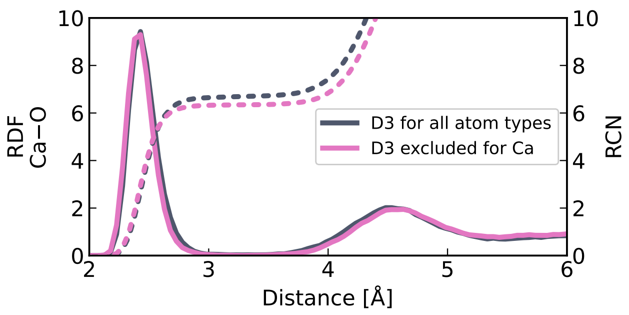

Figure S2: RDFs of calcium–oxygen (full) and corresponding RCN (dashed) including the D3 correction for all atom (grey) and for all kinds but calcium (pink).

plied to the calcium cation, the CN is slightly larger at 6.68 water molecules in the first hydration shell. In contrast, a CN of 6.33 is obtained when the cationic D3 correction is not used. This observation is consistent with the results presented for lithium, sodium, and potassium in the main text.

water structure

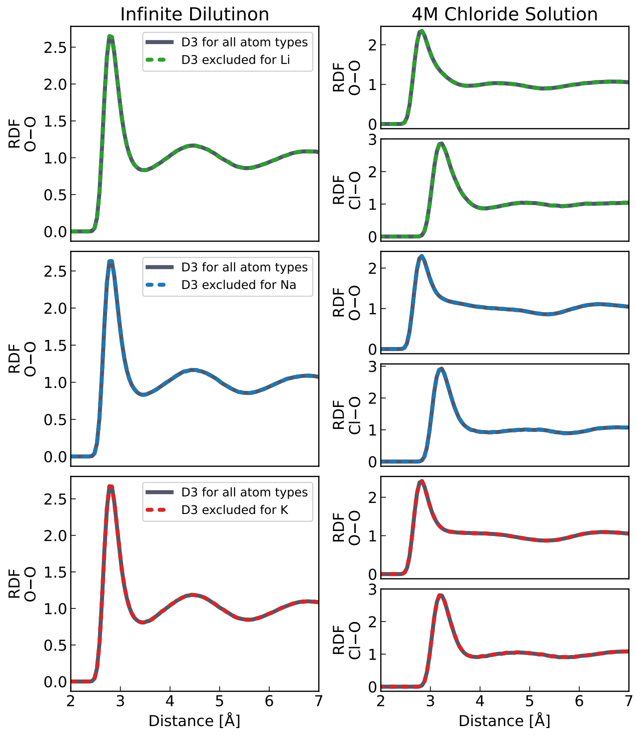

Figure S3: RDFs in the infinitely diluted system (left panels) and in the 4M chloride solution (right panels). Oxygen–oxygen RDFs are displayed for all systems, and oxygen–chloride RDFs only for the 4 M solutions. Cations are color-coded as green (lithium), blue (sodium), and red (potassium).

References

(S1) Kohagen, M.; Mason, P. E.; Jungwirth, P. Accurate description of calcium solvation in concentrated aqueous solutions. *J. Phys. Chem. B* **2014**, *118*, 7902–7909.
