# Supplementary material for: Common Cations Are Not Polarizable: Effects of Dispersion Correction on Hydration Structures from Ab Initio Molecular Dynamics
Source: J Phys Chem Lett. 2023 May 4;14(19):4403–8. doi: 10.1021/acs.jpclett.3c00856 (PMC10201575; doi:10.1021/acs.jpclett.3c00856)
Supplement: Supplementary file 2 — jz3c00856_si_002.pdf [file jz3c00856_si_002.pdf]

jz-2023-00856a.R1

Name: Peer Review Information for "Common Cations Are Not Polarizable: Effects of Dispersion Correction on Hydration Structures from Ab Initio Molecular Dynamics"

First Round of Reviewer Comments

Reviewer: 1

Comments to the Author

The manuscript discusses the use of density functional theory (DFT) approximations for ab initio molecular dynamics of water and solutions of metal cations. To describe the structure and other properties of water, it is generally recommended to use dispersion corrections. However, the accuracy of the corrections depends on the accuracy of the C_6 coefficients and it's known that these are difficult to obtain reliably for atoms with large partial charges, such as the metal cations discussed in the manuscript. The authors show that it's actually more sensible to exclude the cations from the calculation of the dispersion correction. The work nicely shows that using some widely used method blindly without previous testing on one's own system is still not advisable for DFT. The changes are rather significant and relevant for other systems as well. Moreover, the authors show that even the newer "D4" correction is not safe to use for their system, which is also an interesting point. I think the paper is suitable for JPCL after the authors address the following comments.

While D3 doesn't use electron density to obtain the C_6 coefficients, as the authors write, it tries to make them environment dependent. Is there some dependency for the cations or are they really constant?

A reference could be added to substantiate the statement at the end of the second paragraph. The Iterative Hirshfeld Tkatchenko Scheffler scheme should be mentioned as it tries to solve the problem of atomic vs. ionic C_6 coefficients.

On page 2, line 52, in the sentence "It is reassuring ... " the statement is correct only for the CN, the agreement between calculation and experiment doesn't seem to improve for RDF_max when the D3 are switched off for the cations. In Table I it should be made clear what's meant by "(D3)" in the ion column.

In the last paragraph on page 2 it's not clear if water-cation or cation-cation properties are being discussed. The changes in "cation-water RDFs" are explained with the help of "cation-cation" binding curves. The "cation-water" curves would be more appropriate. The paragraph seems to jump from the cation-water discussion into cation-cation.

There are few technical points to fix: missing comma on page 2 line 48, missing space on page 3, line 41, reference 37. Stefan Grimme gets acknowledged twice.

Author's Response to Peer Review Comments:

Dear Editor,

thank you very much for your kind email and the positive referee report. As outlined in detail in the attached file, we have addressed all the points raised by the reviewer, as well as all the editorial issues.

with best regards

pavel

# **Reply to reviews for**:
# Common Cations are not Polarizable: Effects of Dispersion Correction on Hydration Structures from Ab Initio Molecular Dynamics

April 26, 2023

## Editorial changes

We have made modifications to the manuscript suggested by the editor in order to comply with the JPCL formatting guidelines. Specifically, we labeled the abstract section, adjusted the formatting of the references, properly labeled and resized the TOC graphic, and updated the page numbering in the Supporting Information section. Also, the technical remarks brought by the reviewer were corrected.

## Ad Reviewer 1

**Q1:** While D3 doesn't use electron density to obtain the $C_6$ coefficients, as the authors write, it tries to make them environment dependent. Is there some dependency for the cations or are they really constant?

**A1:** The $C_6$ coefficients were specified and optimized for each atom pair, which means that there is some dependence on the local environment. However, this approach is unable to differentiate between chemical environments beyond recognition of individual elements. This means that, for instance, the Na–O interaction will be assigned the same $C_6$ coefficient regardless of whether the oxygen is part of water or sulfuric acid. In other words, although

the $C_6$ coefficients are optimized for each atom pair, they do not take into account the specific chemical environment surrounding the atoms.

**Q2:** A reference could be added to substantiate the statement at the end of the second paragraph. The Iterative Hirshfeld Tkatchenko Scheffler scheme should be mentioned as it tries to solve the problem of atomic vs. ionic $C_6$ coefficients.

**A2:** As the reviewer suggested, we cite the the Iterative Hirshfeld Tkatchenko Scheffler dispersion scheme, which, however, is also not able to capture dispersion for alkali cations correctly.

**Q3:** On page 2, line 52, in the sentence "It is reassuring ... " the statement is correct only for the CN, the agreement between calculation and experiment doesn't seem to improve for $RDF_{max}$ when the D3 are switched off for the cations. In Table I it should be made clear what's meant by "(D3)" in the ion column.

**A3:** In the main text, we clarify that better agreement with experiment is obtained mainly for the coordination numbers, while position of the first peak in the ion–water radial distribution function shifts closer to the experiment for sodium but further away for potassium when the cationic D3 correction is disabled.

We also modified caption of Table 1 such that the "(D3)" label is explained.

**Q4:** In the last paragraph on page 2 it's not clear if water-cation or cation-cation properties are being discussed. The changes in "cation-water RDFs" are explained with the help of "cation-cation" binding curves. The "cation-water" curves would be more appropriate. The paragraph seems to jump from the cation-water discussion into cation-cation.

**A4:** The corresponding part of the text was modified such that it is clear when the cation–cation RDF and when the cation–water RDF is referred to.
